# Blood glucose modulation and safety of efferent vagus nerve stimulation in a type 2 diabetic rat model

**DOI:** 10.14814/phy2.15257

**Published:** 2022-04-19

**Authors:** Sophie C. Payne, Glenn Ward, James B. Fallon, Tomoko Hyakumura, Johannes B. Prins, Sofianos Andrikopoulos, Richard J. MacIsaac, Joel Villalobos

**Affiliations:** ^1^ Bionics Institute East Melbourne Victoria Australia; ^2^ Department of Medical Bionics University of Melbourne Parkville Victoria Australia; ^3^ Department of Endocrinology and Diabetes St Vincent’s Hospital Melbourne Fitzroy Victoria Australia; ^4^ Melbourne Medical School University of Melbourne Parkville Victoria Australia; ^5^ Department of Endocrinology Royal Melbourne Hospital Parkville Victoria Australia; ^6^ Australian Centre for Accelerating Diabetes Innovations University of Melbourne Melbourne Australia; ^7^ Department of Medicine (Austin Health) University of Melbourne Heidelberg Victoria Australia

**Keywords:** autonomic nervous system, bioelectric medicine, directional stimulation, medical devices, metabolic disease, selective peripheral nerve stimulation

## Abstract

Vagus nerve stimulation is emerging as a promising treatment for type 2 diabetes. Here, we evaluated the ability of stimulation of the vagus nerve to reduce glycemia in awake, freely moving metabolically compromised rats. A model of type 2 diabetes (*n* = 10) was induced using a high‐fat diet and low doses of streptozotocin. Stimulation of the abdominal vagus nerve was achieved by pairing 15 Hz pulses on a distal pair of electrodes with high‐frequency blocking stimulation (26 kHz, 4 mA) on a proximal pair of electrodes to preferentially produce efferent conducting activity (eVNS). Stimulation was well tolerated in awake, freely moving rats. During 1 h of eVNS, glycemia decreased in 90% of subjects (−1.25 ± 1.25 mM h, *p *= 0.017), and 2 dB above neural threshold was established as the most effective “dose” of eVNS (*p *= 0.009). Following 5 weeks of implantation, eVNS was still effective, resulting in significantly decreased glycemia (−1.7 ± 0.6 mM h, *p *= 0.003) during 1 h of eVNS. There were no overt changes in fascicle area or signs of histopathological damage observed in implanted vagal nerve tissue following chronic implantation and stimulation. Demonstration of the biocompatibility and safety of eVNS in awake, metabolically compromised animals is a critical first step to establishing this therapy for clinical use. With further development, eVNS could be a promising novel therapy for treating type 2 diabetes.

## Graphical abstract

We demonstrated that our novel vagus nerve stimulation strategy is safe and effective in reducing glycemia, an effect that is repeatable, long‐lasting, and persists despite the continued influence of disease promoting factors, such as a high‐fat diet.

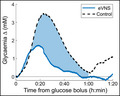

## 1 INTRODUCTION

Diabetes mellitus is a chronic, progressive condition that affects over 420 million people worldwide (WHO, 2017), with total cost of treatment estimated to be $327 billion in 2017 (ADA, 2018). The principal defects of the disease involve insulin resistance, and progressive pancreatic beta cell dysfunction leading to the deterioration of glycemic control (Stumvoll et al., 2005) and serious secondary complications (Zheng et al., 2018). Despite advancements in pharmacological therapies, 45% of patients in the US fail to achieve adequate glycemic control (<7% HbA1c) (Brown et al., 2010; EMA, 2012; Harrower, 1994; Wang et al., 2021). Bionic medicine is used to alter the activity of peripheral nerves to treat conditions and is emerging as a promising treatment for many chronic diseases (Alliance for Advancing Bioelectronic Medicine, 2020; Malbert, 2018; Vitale & Litt, 2018). Electrical modulation of the vagus nerve to lower high glucose levels has been the focus of bionic medicine research (Guemes Gonzalez et al., 2020; Malbert, 2018) as this autonomic nerve is involved in the regulation of food intake, glucose metabolism, and homeostasis and influences the overall dynamics of insulin secretion (Travagli & Browning, 2011; Waise et al., 2018). However, exactly how to modulate the activity of the vagus nerve to achieve optimal, glycemic control is yet to be determined.

Rat studies show that stimulating the distal cut end of the cervical vagus nerve, thereby selectively activating efferent (preganglionic motor) fibers, causes a sustained increase in insulin, suppression of glucagon and the eventual reduction in blood glucose levels (Meyers et al., 2016). Direct electrical stimulation of preganglionic (efferent) vagal neurons resident in the dorsal motor nucleus (DMN) increases insulin levels (Ionescu et al., 1983). Furthermore, pharmacological activation and disinhibition of discrete subpopulations of vagal DMN neurons induced by a glutamate agonist or GABA_A_ receptor antagonist, respectively, both result in the increase of pancreatic exocrine secretions in rats (Babic et al., 2012; Mussa & Verberne, 2008). Therefore, an overarching aim of this study was to evaluate the effectiveness of vagus nerve stimulation designed to preferentially induce efferent conduction of vagus activity (eVNS) to reduce glycemia in a rat model of type 2 diabetes.

Reversible, selective stimulation of the efferent (or “distal”) nerve bundle within a peripheral nerve can be achieved by combining electrical kilohertz‐frequency blocking with low‐frequency activation (Patel et al., 2017). Although the cervical vagus nerve is often the target site of stimulation, unpleasant off target effects to breathing and heart rate limit the amount of stimulation that can be tolerated in humans (Payne et al., 2019). However, the abdominal vagus is an ideal stimulation site as the nerve is distal to vagal branches that innervate the heart and lungs, bypassing thoracic off target effects during stimulation (Payne et al., 2019). Previously, we showed eVNS was successfully applied to the abdominal vagus nerve of normal anesthetized rats and prevented the hyperglycemic effects seen following standard vagus nerve stimulation (15 Hz, VNS) (Payne et al., 2020). As such, we hypothesized that abdominal eVNS would reduce hyperglycemia in a model of type 2 diabetes. Second, we hypothesized that chronic implantation and eVNS would be tolerated and histologically safe when applied to awake, freely moving but metabolically compromised rats. To address these hypotheses, we used our custom cuff electrode array designed for chronic implantation onto the abdominal vagus nerve of awake rats (Payne et al., 2019). The optimal “dose” of eVNS to reduce glycemia was evaluated in awake rats following an oral glucose tolerance test. The effectiveness and stability of eVNS were assessed after the chronic implantation period in anesthetized rats by an intravenous glucose tolerance test. The tolerability and safety of chronic implantation and stimulation (1 h/day, over 3 weeks) were assessed by histologically examining the abdominal vagus nerve.

## 2 MATERIALS AND METHODS

### 2.1 Animals

Procedures were approved by the St. Vincent's Hospital (Melbourne) Animal Ethics Committee (Project/approval number: 025–18) and complied with the Australian Code for the Care and Use of Animals for Scientific Purposes (National Health and Medical Research Council of Australia) and the Prevention of Cruelty to Animals (1986) Act. All experiments used male Sprague‐Dawley rats (5 weeks old at the start of the experiment). Animals were kept on a 12‐h light/dark cycle and were fed ad libitum. Rats were kept two to a cage initially, and were single housed postimplantation, in clear walled cages and provided with environmental enrichment. For all surgical procedures, rats were anesthetized in 1.5%–3% isoflurane (1 L/min oxygen) and given preoperative analgesic (0.3 mg/kg Temgesic, subcutaneous injection). On the day of sacrifice, rats were euthanized using 300 mg/kg Lethabarb (intramuscular injection).

### 2.2 Type 2 diabetes model

**FIGURE 1:**
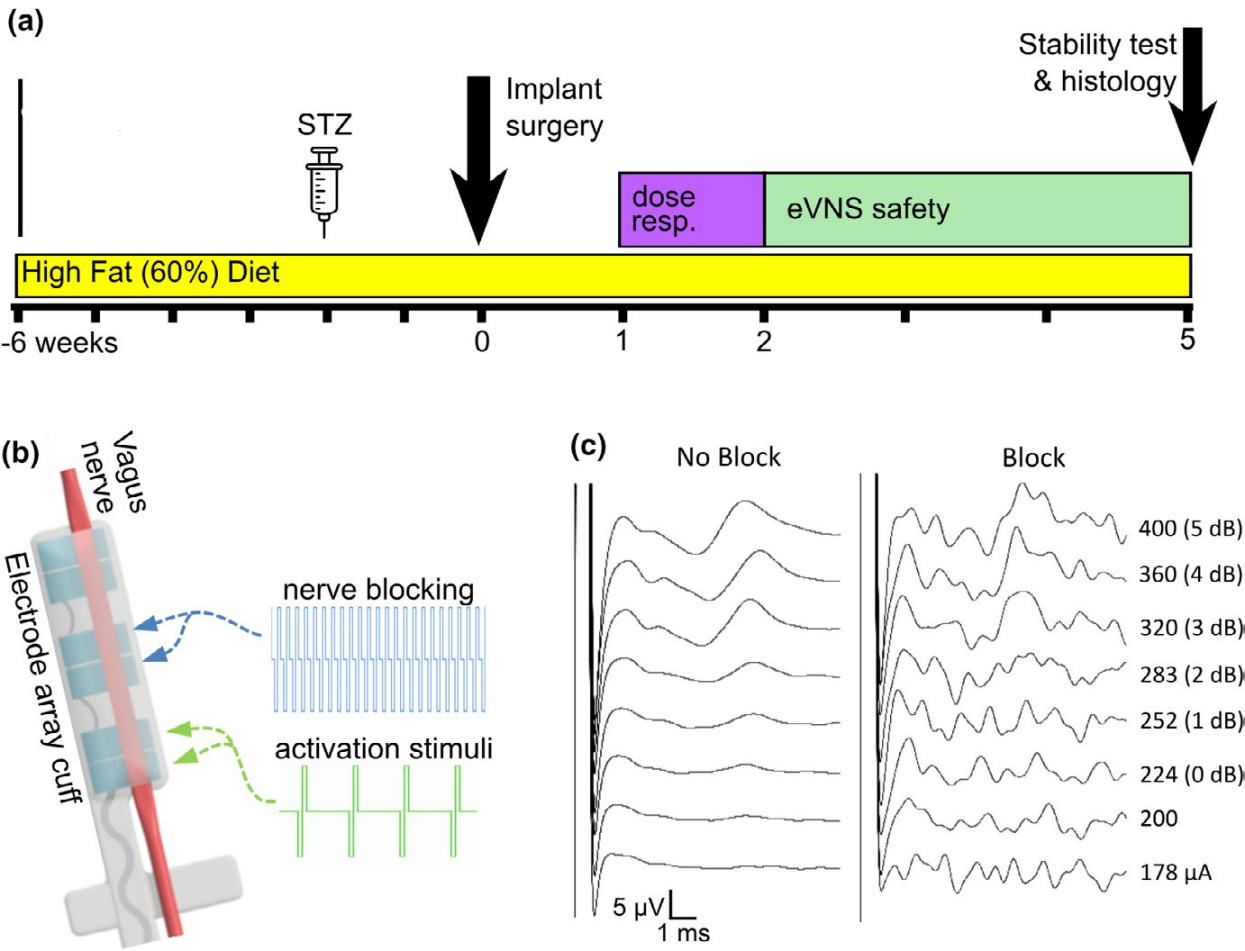
Experimental schedule and the efferent vagus nerve stimulation strategy (eVNS). (a) Experimental schedule outlining the rat model and eVNS testing. (b) Schematic drawing of the abdominal vagus nerve cuff electrode array. The middle electrode pair (E3‐E4) provided blocking stimulation (26 kHz) and the distal electrode pair (E5‐E6) delivered an activation stimulation (15 Hz). The proximal electrode pair (E1‐E2) were used to record evoked compound action potentials (ECAPs). (c) Illustrative ECAP recordings from an acutely implanted, metabolically challenged rat (not used in this study) during no block (224 µA threshold) and during 26 kHz blocking

Rats were fed a high‐fat diet (HFD, 60% fat, Specialty Feeds, SF03‐002) for the duration of the experiment (Figure 1a). After 5 weeks on the diet, rats were anesthetized (1.5%–3% isoflurane, 1 L/min oxygen) and a low dose of streptozotocin (STZ, 35 mg/kg intraperitoneal in citrate buffer, pH 4.5) given (Skovso, 2014; Yin et al., 2019). One week later, rats were given a second STZ injection if fasted blood glucose levels remained within a normal range (rats 30, 32, 33). Tail vein fasted blood glucose was taken and measured using a glucometer (Accu‐Chek Nano, Roche). Animals were included in the study if fasted blood glucose was ≥10 mM or random blood glucose ≥12 mM within 1 week of STZ injection but were excluded if blood glucose levels were ≥30 mM or showed type 1 diabetic symptoms (e.g., sudden weight loss). As a secondary qualitative indicator of the disease, rats included in the study were observed to have polyuria and increased water consumption. Two animals (out of 13 injected with STZ) had high blood glucose levels (>30 mM), were euthanized immediately and excluded from the study. No animals died before meeting the criteria for euthanasia, and the remaining 11 animals that showed type 2 diabetic symptoms were monitored daily for the duration of the experiment (5 weeks).

### 2.3 Vagus nerve electrode array design

As described previously (Payne et al., 2020), the vagus nerve electrode array (Figure 1b) consisted of three‐pairs of platinum (99.95%) electrodes (0.39 mm^2^ surface area, 3.4 mm distance between electrode pair sites) in a silicone substrate. A rectangular channel (0.55 mm × 0.2 mm) traversed the cuff to accommodate the vagus nerve, and the cuff was sutured closed to keep the nerve resident within the channel. The array was anchored to the esophagus to provide mechanical stability and a lead tunnelled to a percutaneous connector mounted on the lumbar region of the rat.

### 2.4 Implantation surgical procedures

After 2–4 weeks following STZ injection, rats were given preoperative analgesic (0.3 mg/kg Temgesic, S.C), anesthetized (1.5%–3% isoflurane, 1 L/min oxygen) and prepared for aseptic surgery (Figure 1a). Vagus nerve implantation surgery: Similar to that described previously (Payne et al., 2020), the abdominal cavity was exposed and the electrode array implanted onto the anterior sub‐diaphragmatic abdominal vagus nerve, above the hepatic, celiac, and gastric vagal branches. Telemetry implantation surgery: During the same surgery, a fully implantable glucose telemetry sensor (HD‐XG, Data Sciences International) was inserted into the abdominal aorta. The telemetry electronics capsule was sutured on the internal wall of the abdominal cavity. At the conclusion of the surgery the abdominal cavity was sutured shut and the percutaneous pedestal (of the vagus nerve electrode array) sutured to the dorsal lumbar aspect of the animal. Animals were given appropriate postoperative care and allowed to recover. Three animals (out of 13 surgeries) were excluded and euthanized early due to anesthetic complications, high blood glucose (see above), and vascular complications related to the telemetry sensor. The final cohort included *n* = 10 animals.

### 2.5 Impedance testing and evoked neural recordings

Electrical impedance: Functionality of electrodes was tested post‐surgery, prior to eVNS on the day of termination. Impedance was measured from end‐of‐phase voltage transients during current pulses (100 µs phase width, 25 µs interphase gap at 100 µA current), in common‐ground configuration (one active electrode vs. all others as return) (Fallon et al., 2009). Evoked neural recordings: Electrically evoked compound action potentials (ECAPs) were also recorded post‐surgery, after 1 week and on the day of termination. To determine ECAP threshold, the distal electrode pair (closest to the pancreas/liver) was stimulated with bi‐phasic current pulses (200 µs; 15 Hz; 0–2 mA) and evoked activity was recorded from the proximal pair of electrodes (averaged over 200 pulses) using an isolated differential amplifier with active probe (ISO‐80. World Precision Instruments). Recordings were sampled at a rate of 200 kHz and digitally filtered (200–2,000 Hz band pass) using a data acquisition device (USB‐6210, National Instruments) (Fallon & Payne, 2020; Payne et al., 2019). The threshold of evoked neural responses (acquired without blocking stimuli) was visualized using IGOR Pro‐8 and defined as the minimum stimulus intensity producing a response amplitude above noise (at least 0.1 μV) within a post‐stimulus latency window of 4–10 ms (Fallon & Payne, 2020).

### 2.6 Vagus nerve stimulation therapy

The stimulation method aimed to activate the efferent (distal) nerve bundle and apply a focal block at the electrode site to produce predominantly directional stimulation, a method that has been validated previously (Payne et al., 2020). A custom stimulator (Fallon, 2018) was programmed to deliver 26 kHz stimulation (bi‐phasic current pulses at 4 mA, 10 µs/phase) to the central electrode pair and 15 Hz (200 µs bi‐phasic square pulses) to the distal electrode pair (Payne et al., 2020) (Figure 1b). The battery‐operated stimulator was designed to deliver constant current pulses up to 4 mA using a maximum of 25 V in 16 channels. The pulses had leading cathodic phase followed by an 8 μs gap before an anodic phase for charge recovery, and the electrodes were shorted between stimulation pulses. As described previously (Payne et al., 2020), eVNS can be defined from a change in the activating current level that evokes neural activity while 26 kHz blocking stimulation is applied (Figure 1b,c). The current level applied to the distal electrode pair delivering 15 Hz activating stimulation was examined in this study and was referred to as the eVNS “dose”. The dose of eVNS is measured in decibels (dB) and refers to the amount of current given above the ECAP threshold (Figure 1c). For example, an eVNS dose of 2 dB refers to a current level set at 2 dB higher than the ECAP threshold. Representative ECAPs during blocking and no blocking in Figure 1(c) were recorded from a metabolically challenged rat (ID: 20, fasted blood glucose: 11.1 mM) not included in the rest of this study.

### 2.7 Dose‐response testing of eVNS (awake)

At 1 week following implantation surgery, animals were fasted for 3–4 h and oral glucose tolerance tests (OGTT) was performed on awake rats by oral gavage of a bolus of glucose (500 mg/kg) and continuous recording of blood glucose via the glucose telemetry system. Multiple dose response tests were repeated daily to evaluate the response to a series of eVNS current levels. In two animals (24 and 26) there were additional tests for 2 weeks. Immediately after the administration of the glucose bolus, rats received eVNS for exactly continuous 60 min at 0.5, 2, 3, 4, or 6 dB above ECAP threshold. The response was compared to the unstimulated (control) test, which was repeated in the same rat at the beginning and end of the week. The response was quantified from continuous glucose readings, by obtaining the area under the signal over 60 min minus baseline and subtracting from the mean area of the unstimulated control test readings.

### 2.8 Safety testing of eVNS (awake)

To assess tolerability and electrical safety of chronic eVNS, rats were stimulated for 60 min a day over 3 weeks using a current level of 2 dB above ECAP threshold (Figure 1a,c).

### 2.9 Stability of eVNS following chronic implantation (anesthetized)

After 5 weeks of implantation and stimulation, rats were subjected to a final non‐recovery anesthetized experiment to study the efficaciousness of eVNS (Figure 1a). Stimulation was applied for 1 h following an intravenous bolus of glucose, similar to that described previously (Payne et al., 2020). In brief, animals were anesthetized (1.5%–3% isoflurane, 1 L/min oxygen) and the femoral veins cannulated to deliver an intravenous bolus of glucose (300 mg/kg, T = 0) and withdraw serial blood samples (300 µl; T = −5, 5, 12, 30, 60, 90 min) for analysis of hormones. eVNS was applied for 60 min from the application of glucose bolus, and no stimulation was used as a control (Payne et al., 2020).

### 2.10 Blood glucose calibration

The implantable glucose telemetry sensor (HD‐XG, Data Sciences International) was calibrated according to the manufacturer's instructions using the Single Point Calibration method. In brief, calibration was conducted at least twice a week, at a similar time each day, immediately prior to an oral glucose tolerance test. Rats were fasted for 14 h and tail vein fasted blood glucose was measured using a glucometer (Accu‐Chek Nano, Roche), which was previously validated and found to be highly correlated with a glucose assay kit (Pearson *R* ^2 ^= 0.85; kit 81693, Crystal Chem) (Payne et al., 2020).

### 2.11 Molecular analysis of hormones

Whole venous blood samples (300 µl) were collected (K2‐EDTA coated tubes, Sarstedt), centrifuged (2,000 ***g*** for 10 min) and extracted plasma stored at −80°C. On the day of the assay, aliquots were thawed and enzyme‐linked immunosorbent assays (ELISA) for insulin (kit: 90010; Crystal Chem) and glucagon (kit: 81519) performed according to manufacturer's instructions. Hormone levels were determined via absorbance measurements using a VICTOR^®^ Nivo^™^ Multimode Microplate Reader (PerkinElmer, Inc).

### 2.12 Vagus nerve dissection and histology

At the conclusion of the experiment, rats were euthanized (300 mg/kg Lethabarb, intramuscular injection) and transcardially perfused (0.9% saline, 10% neutral buffered formalin). Implanted tissue was dissected, post‐fixed overnight at 4°C and washed thoroughly (phosphate buffered saline, pH 7.4). The implanted vagus nerve adjacent to the electrodes E1–E2 vs. E3–E4 vs. E5–E6 was labelled using tissue dye (Davidson's Marking system, Bradley Products) (Payne et al., 2019). Tissue proximal to the implanted site was also taken. The esophagus and attached vagus nerve were frozen in optimal cutting temperature compound (−20°C, ProSciTech), serial sections (10 µm) taken. Sections were stained with hematoxylin and eosin (H&E, Sigma) and mounted with dibutyl phthalate polystyrene xylene (DPX). Tissue from the implanted nerve was examined by a blinded observer (S.C Payne) for signs of inflammation and damage (Payne et al., 2019). At each location light microscope images were taken using a Zeiss Axioplan II microscope and Axiovision software (Zeiss). Total fascicle cross‐sectional area was quantified across one representative section per electrode position per animal, using Fiji (Open Source image processing and analysis software, Fiji.sc).

### 2.13 Statistical analysis

For the glucose tolerance testing data, the area under the curve using glycemia change from baseline (glycemia δ) was calculated over the 60‐min testing period. Due to missing values, a one‐way anova was used to assess data over multiple current amplitude levels. The response to stimulation during acute eVNS was calculated as the area of this glycemia δ, minus the mean of the area of glycemia δ without stimulation (averaged from two recordings). The effect of eVNS on the levels of glucose, glucagon, and insulin were compared to the internal unstimulated response using paired Student's *t* tests or repeated measures anova to assess differences. For each hormone measurement, the baseline at T = −5 min was subtracted and the area under the curve calculated over the 60‐min period during which stimulation was applied. The fascicle area was assessed using a repeated measures one‐way anova. Statistically significant differences were accepted as *p *< 0.05 and Minitab software or GraphPad Prism 4 (GraphPad Software) used for all analysis.

## 3 RESULTS

### 3.1 Efficacy of eVNS

#### 3.1.1 Impedance and electrically evoked neural response thresholds

Immediately following surgery, impedances were tested (E1‐E2: 7.0 ± 0.6 kΩ. E3‐E4: 6.7 ± 0.6 kΩ; E5‐E6: 8.5 ± 0.8 kΩ) and 9 of 10 implants were fully functional (Table 1). At the time of termination, there were no significant changes (one‐way RM anova, Time: *p *= 0.311, *n* = 9) in impedances compared to post‐surgery values. Furthermore, there were no significant differences (*p *= 0.251) in the mean impedance between stimulating (E1‐E2: 8.6 ± 1.05 kΩ), blocking (E3‐E4: 7.1 ± 1.25 kΩ) and unstimulated electrodes (E5‐E6: 9.6 ± 0.97 kΩ) following chronic implantation and stimulation. In animal 27, two electrodes were shorted so this animal was excluded from all eVNS testing but included in the histological analysis to assess the effects of chronic implantation (Table 1). Animal 30 had functional electrodes but was presumed not to have received stimulation due to a postmortem analysis showing the nerve had slipped out of the electrode‐interface channel (Table 1). Animal 24 had its percutaneous connector extruded and surgically removed 4 weeks after implantation, and so was excluded from a final glucose tolerance test (under anesthesia). All the electrodes in stimulated animals (*n* = 8) remained functional for the duration of implantation.

**TABLE 1:** Summary of chronically implanted animals

Animal ID	Implantation time (weeks)	Postoperative ECAP threshold (µA)	Final ECAP threshold (µA)	Total eVNS time (h)	Chronic Stimulation	Comments
24	7	420	Not tested	18	Yes	Dorsal connector lost on week 4
26	7	320	500	27	Yes	
27	7	Invalid	Invalid	6	No	Shorted electrodes
30	6	None	None	3	No	Nerve slipped from electrode cuff
32	6	None	None	13	Yes	
33	5	400	710	20	Yes	
34	5	450	500	20	Yes	
35	4	400	500	14	Yes	
36	5	None	None	18	Yes	
39	4	480	1,000	15	Yes	Infection around dorsal connector

Prior to eVNS dose‐response testing, ECAPs were recorded and the threshold was found to average 412 ± 22 µA (Table 1). At the end of the implantation period, the mean ECAP thresholds were 642 ± 98 µA (paired *t* test, *p *= 0.0513).

#### 3.1.2 Dose‐response testing of acute eVNS

**FIGURE 2:**
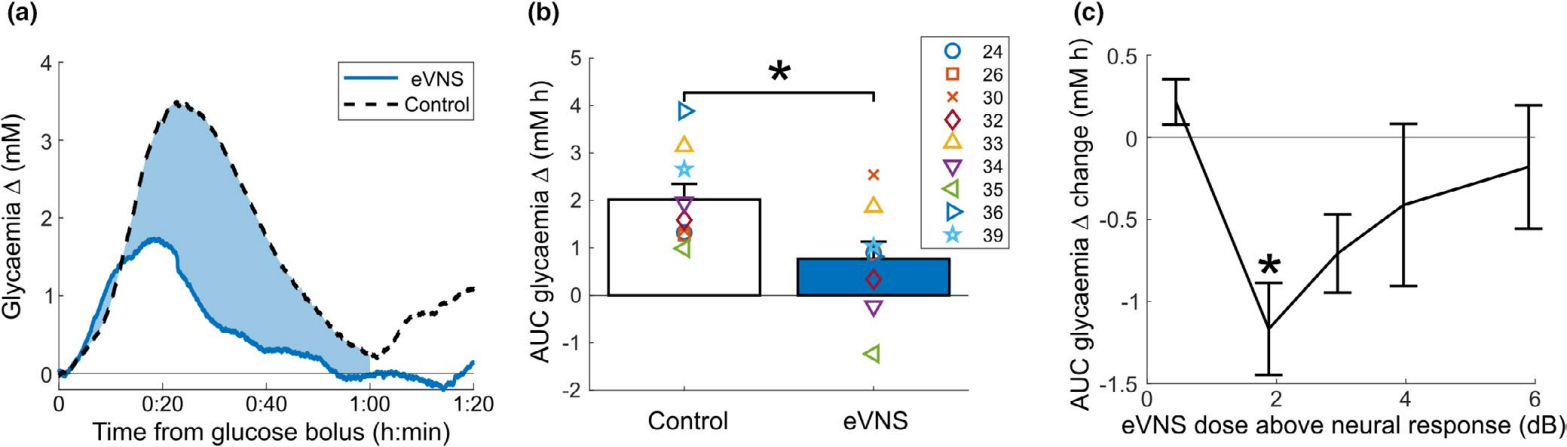
Dose effects of acute eVNS during an oral glucose tolerance test. (a) Representative example shows a reduction (indicated by blue shading) in glycemia during 2 dB eVNS (1 h, blue line) compared to control (no stimulation, dotted black line). (b) Changes in the area under the curve (AUC) of glycemia during acute eVNS compared to internal unstimulated control. (c) Changes in glycemia AUC when different current levels above ECAP threshold were applied. Data were compared to the control glycemic response within the same animal and show the difference in glycemia from baseline (T = 0). Data in (b,c) show mean ± *SEM*, symbols are individual subject values, and significance was accepted as values *p *< 0.05 (indicated by “*”)

Following high‐fat diet and STZ injection, fasting blood glucose of rats increased from 4.80 ± 0.18 mM to 11.64 ± 2.28 mM (paired *t* test, *p *= 0.015, *n* = 9). The effect of acute eVNS (1 h) was assessed at various current amplitudes in awake rats during an OGTT (Figure 2a). In eight out of nine rats, the glycemic AUC response was reduced by 64 ± 82% (1.25 ± 1.25 mM h, paired *t* test, *p *= 0.017) during eVNS, compared to unstimulated control (Figure 2b). In a subset of rats (*n* = 6) in which ECAPs were confirmed (Table 1), thereby confirming vagal activation, all rats responded to eVNS. When eVNS was delivered with a current 2 dB above ECAP threshold, the glycemic AUC significantly decreased by −1.2 ± 0.28 mM h (−72 ± 76%), compared to unstimulated control (one‐way anova, Dunnett's post hoc test, *p *= 0.009, *n* = 6). However, there were no differences (*p *≥ 0.05) in glycemic AUC when 0.5 dB (+0.22 ± 0.22 dB, *n* = 2), 3 dB (−0.71 ± 0.24 dB, *n* = 4), 4 dB (−0.41 ± 0.5 dB, *n* = 5), and 6 dB (−0.18 ± 0.38 dB, *n* = 6) were applied, compared to unstimulated control (Figure 2c). As such, a stimulation current or eVNS “dose” of +2 dB above the ECAP threshold was used for all subsequent experiments described below. The extent of the therapeutic window was quantified using the half‐width of the response curve for each animal and was on average 2.4 ± 1.2 dB.

#### 3.1.3 Chronic stability of acute eVNS following chronic implantation

**FIGURE 3:**
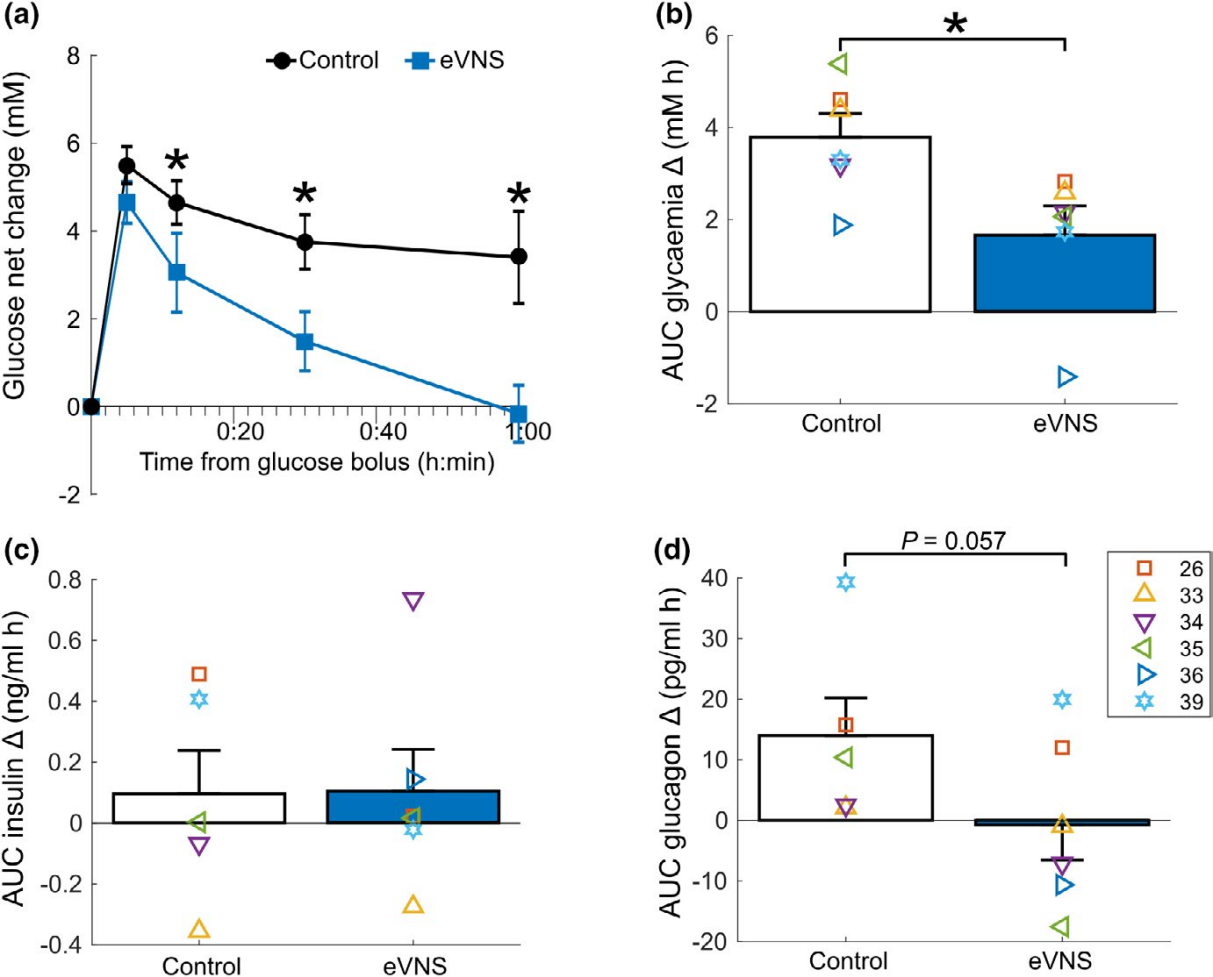
Chronic stability of eVNS during an oral glucose tolerance test following chronic implantation. (a) Average net changes in glucose (*n* = 6), compared to baseline, during 60 min of 2 dB eVNS and no stimulation (control). (b) Comparison of the glycemic AUC during 1 h of eVNS and no stimulation (control). (c) Comparison of the insulin AUC during eVNS and no stimulation (control). (d) Comparison of the glucagon AUC during eVNS and no stimulation (control). Data in (b–d) show AUC of the change from baseline (T = 0). Graphs show mean ± *SEM*, symbols represent individual subject values, and significance was accepted as values *p *< 0.05 (indicated by ‘*’)

Following chronic implantation and stimulation, the acute (1 h) effect of eVNS (+2 dB above ECAP threshold) was assessed during an intravenous glucose tolerance test under anesthesia. Net change in glucose (from baseline) was significantly (RM two‐way anova, Time: *p *< 0.0001, Treatment *p *= 0.003) less at 12 min (*p *= 0.035), 30 min (*p *= 0.002), and 60 min (*p *< 0.0001, Figure 3a). Analysis of the AUC glycemic change shows an overall decrease of 56% during eVNS, compared to unstimulated control (−1.7 ± 0.6 mM h, paired *t* test: *p *= 0.003) compared to the unstimulated control (Figure 3b). There were no changes in insulin levels during eVNS (95 ± 157 pg/ml h) compared to unstimulated control (96 ± 169 pg/ml h; *p *= 0.997, Figure 3c). However, there was a trend decrease in glucagon levels during eVNS (1.3 ± 6.68 ng/ml, *p *= 0.057) compared to unstimulated control levels (14.0 ± 6.82 pg/ml, Figure 3d), and the reduction in glucagon between eVNS and control correlated with the reduction in glycemia (*R* ^2 ^= 0.66).

### 3.2 Safety of eVNS in metabolically compromised rats

#### 3.2.1 Tolerability of chronic implantation and stimulation

##### Implantation

There were no unexpected deaths specifically related to the implantation of the vagus nerve electrode array. Rats were implanted for an average of 5.6 ± 1.2 weeks (range: 4–7 weeks). The abdominal surgical incision site healed quickly, and in nine out of 10 rats, the tissue in proximity with the subcutaneous cable remained free from infection and seromas. The skin surrounding the percutaneous pedestal was occasionally irritated and required higher levels of cleaning and maintenance compared to normal rats. Rat 39 sustained a chronic infection that manifested around the cable and around the outside of the electrode cuff, likely related to a breach in sterility during implantation surgery. Despite these complications, the animal continued to gain similar amounts of weight to that seen in other animals, and ECAPs were successfully generated prior to termination (Table 1).

##### Stimulation

During dose‐response and chronic stimulation experiments, eVNS was delivered (*n* = 8, Table 1) for a mean total of 16.6 ± 2.1 h (range: 13–27 h, Table 1). Variation in total stimulation time between subjects is due to a varying number of dose‐response tests given and the loss of a connector in one subject (rat 24, Table 1). No adverse responses, such as startling, agitation, or coughing, were observed following the onset of eVNS, and rats usually displayed normal grooming and exploratory behavior. Furthermore, the weight of rats that received 3 weeks of chronic eVNS continued to increase over the 3‐week stimulation period (start of eVNS: 425 ± 12.8 g to 470 ± 12.0 g; paired *t* test *p *= 0.0003, *n* = 6). Weight of unstimulated (age‐matched) rats at a comparable time‐period increased from 410 to 515 g (rat 27) and 317 to 349 g (rat 30).

#### 3.2.2 Glycemic changes during eVNS safety testing

In stimulated rats (Table 1), fasted blood glucose increased from 7.5 ± 1.5 mM to 9.3 ± 2.0 mM during the 3‐week testing period, resulting in a significant (paired *t* test, *n* = 6, *p *= 0.044) increase in glycemia of +1.1 ± 0.5 mM. There were no differences in fasted insulin levels between the start (1.4 ± 0.6 ng/ml) and end of the eVNS testing period (0.6 ± 0.3 ng/ml, paired *t* test: *p *= 0.40) and glycated hemoglobin (start: 7.8 ± 1.9%, end: 8.0 ± 1.1%, *p *= 0.39). In rats that could not be stimulated due to issues at the electrode interface (shorting or the nerve slipping out of the electrode channel) (*n* = 2, Table 1), fasted blood glucose increased from 9.3 ± 3.2 mM to 11.5 ± 5.4 mM in the same period. However, no conclusion can be drawn from these data as unstimulated controls were not appropriately powered.

#### 3.2.3 Histopathology following chronic implantation and stimulation

**FIGURE 4:**
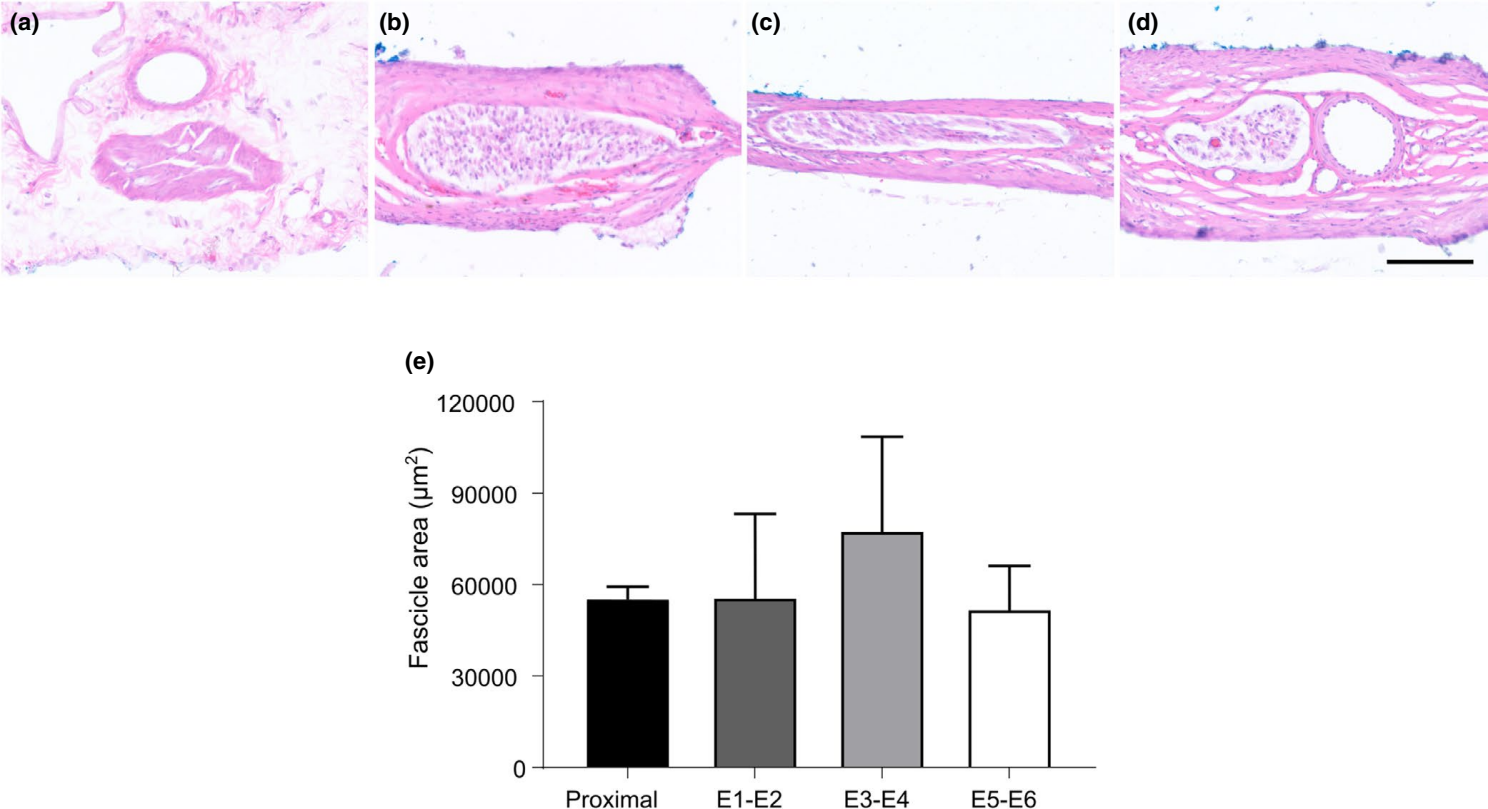
Histological assessment of the vagus nerve following chronic stimulation and implantation. Hematoxylin and eosin staining was used to assess histopathology in neural tissue proximal (a) to the implant, at E1‐E2 (b), E3‐E4 (c), and E5‐E6 (d). (e) The cross‐sectional area of anterior vagus nerve fascicles was analyzed proximal to the implantation site and in tissue adjacent to electrode pairs. Scale bar for (a–d) represents 100 µm and data show mean ± *SEM*, with significance accepted as values *p *< 0.05

In all vagus nerves assessed (*n* = 8 stimulated, *n* = 2 unstimulated, Table 1), tissue taken proximal to the implantation site appeared normal (Figure 4a), with no signs of Wallerian degeneration. Tissue taken from unstimulated animals (27, 30, Table 1) had a mild, benign fibrotic tissue response at all electrode positions. No disruptions to blood vessels, morphological changes to the nerve architecture or infiltration of inflammatory cells were observed. Old hematomas within the exterior tissue encapsulation were evident, likely as a result of the initial implantation surgery. Furthermore, the vagus nerve from animal 30 had slipped out of the channel at electrode position E1‐E2, but as this subject did not receive eVNS, this incident did not impact on the delivery of eVNS.

The histopathology of stimulated rats (*n* = 8, Table 1) was assessed. Unstimulated tissue (E1‐E2) had a mild foreign body response with no signs of inflammation or morphological changes to fascicle architecture (Figure 4b). Neural tissue that received high‐frequency blocking stimulation (E3‐E4: 26 kHz at 4 mA) had a mature, extensive but benign foreign body response with little infiltration of inflammatory cells and no granulation (Figure 4c). The appearance of the inferior phrenic artery often appeared smaller or distorted, due to the compaction of fibrotic tissue within the cuff. Although a few samples (*n* = 2) showed indications of old hematomas within the exterior tissue encapsulation, no erythrocytes were observed in tissue adjacent to or within neural tissue. In some animals (32, 33, 34), localized elongation of fascicles were observed and led to a degree of displacement of the nerve from the electrode interface (rats: 33, 34, Table 1; Figure 4c). Neural tissue adjacent to electrodes that received activation stimuli (E5‐E6, 15 Hz at 0.4–0.6 mA) had a mild, benign fibrotic response, with minimal infiltration of acute inflammatory cells, and well preserved inferior phrenic artery architecture (Figure 4d).

In rat 39, an abscess was observed outside of the cuff electrode, close to esophagus, and granulation was observed within implanted neural tissue from each electrode level. Acute inflammatory cell infiltration and granulation were observed within the fibrotic response surrounding the epineurium. However, neural tissue taken proximal to the implant remained free from inflammation.

Quantitative assessment of fascicle area of stimulated rats (*n* = 8, Table 1) did not show evident changes between tissue locations (one‐way RM anova: *p *= 0.772, Figure 4e).

## 4 DISCUSSION

The vagus nerve is involved in the regulation of glucose metabolism and homeostasis and has therefore been the focus of recent bionic neuromodulation research (Guemes Gonzalez et al., 2020; Malbert, 2018). Here, we demonstrate for the first time in awake, freely moving animals paired vagus nerve stimulation with high‐frequency blocking that activates efferent (distal) vagal pathways in metabolically compromised rats. eVNS was most effective at acutely lowering glycemia when applied at 2 dB above the ECAP threshold, and even remained effective at improving metabolic control at the end of the implantation period. Chronic delivery of eVNS was well tolerated in awake, freely moving animals and incurred no histopathological damage to chronically stimulated vagal tissue. Taken together, eVNS is well tolerated, safe and improves metabolic control in metabolically compromised rats.

A growing number of studies have demonstrated directional activation of peripheral nerves in anesthetized rats. Differential fiber‐specific block was first achieved in the sciatic nerve by blocking neural pathways using kilohertz electrical stimulation (20 or 40 kHz, 1.5 mA) (Patel & Butera, 2015). Later, selective activation of the efferent nerve bundle in the cervical vagus nerve was demonstrated in anesthetized rats challenged with a model of sepsis and shown to be more effective at reducing systemic levels of the pro‐inflammatory cytokine TNF‐alpha than regular vagus nerve stimulation (Patel et al., 2017). More recently, combining an anodal block with cervical VNS effectively achieved directional activation of efferent or afferent vagus nerve bundle (Ahmed et al., 2020). Here, we used kilohertz frequency pulsatile electrical stimulation (26 kHz 4 mA, 10 µs/phase) to reduce or block activity. Application of low levels of stimulation (15 Hz, 2 dB above threshold) to distal electrode pair would likely evoke gross directional propagation of the efferent vagus nerve bundle (Payne et al., 2020), which includes efferent (orthodromic) and afferent (antidromic) fibers. It is worth noting that our pulsatile kilohertz block does not produce a complete block as activity was still able to propagate past the block at higher current levels (see Figure 1c). However, when 2 dB above threshold eVNS was applied, it is likely the balance between efferent and afferent activity was altered to have the desired, consistent effect of lowering glucose.

Other studies have applied “regular” VNS (5 Hz) to the cervical vagus nerve of awake rats to assess the effects on glycemia (Stauss et al., 2018). However, to our knowledge this is the first time that our eVNS stimulation strategy has been applied to the abdominal vagus nerve of awake, freely moving metabolically compromised animals, which experienced no adverse reaction to the onset of stimulation and continued to gain weight during testing. By targeting the abdominal vagus nerve, we have overcome potential side effects to breathing and heart rate likely experienced at the cervical vagus nerve. Furthermore, the design of the electrode array, percutaneous plug and custom stimulator allows for the viable translation of this stimulation protocol into awake, freely moving animals. As such, we have overcome anatomical and technological limitations and demonstrated that eVNS was well tolerated in metabolically compromised animals, which is a key step toward clinical translation.

Here, we used a well‐established model of type 2 diabetes (Skovso, 2014), involving a combination of low doses of STZ and a continuous high‐fat diet and that leads to a compromised metabolism. While no histopathology was performed to quantify beta cell loss, all animals had a fasted glucose level of 10 mM or higher but did not display typical symptoms of type 1 diabetes, such as sudden weight loss. As a secondary qualitative indicator of the disease, all rats were observed to have polyuria and increased water consumption.

In eight out of nine of these metabolically compromised animals continuous 1 h of eVNS lowered glycemia by 62% following an oral glucose tolerance test. This is a high response rate to the eVNS, especially since the non‐responding animal (rat 30) was later confirmed as a failure of electrode surgical placement around the nerve. Notably, eVNS dosing had a J‐shaped effect on glycemia, with 2 dB above ECAP threshold being the most effective dose in lowering glycemia, while higher eVNS doses (6 dB) produced no significant change in glycemia. Our preferred explanation is that higher nerve activation currents overcome the focal block, thereby allowing activation of the (orthodromic) vagal afferent pathway toward the brain.

The balance between afferent and efferent activity is likely to be critical in regulating glycemia. Previously we showed that simple 15 Hz VNS and afferent VNS results in a significant increase in blood glucose and glucagon (Payne et al., 2020). A similar observation was seen during cervical VNS in awake, normal rats (Stauss et al., 2018). This afferent‐mediated hyperglycemic effect is likely mediated by the activation of glucose sensing hepatic afferent fibers (Niijima, 1982, 1983), and the suppression of insulin release, centrally mediated by the indirect activation of the sympathetic splanchnic nerve (Andersson et al., 1982; Dunning et al., 1988; Holst et al., 1986; Meyers et al., 2016; Payne et al., 2020). As such, using blocking‐stimuli to limit evoked vagal afferent activity and prevent the hepatic afferent‐mediated hyperglycemic reaction is a key objective for developing an appropriate therapeutic strategy.

Strikingly, glycemia was effectively reduced during 1 h of continuous 2 dB eVNS both at 1 and 5 weeks (72% and 54% reduction, respectively) following implantation, suggesting the delivery of eVNS is stable and not diminished by the tissue response to chronic implantation. eVNS likely acts by the suppression of glucagon (Babic et al., 2012; Meyers et al., 2016; Mussa & Verberne, 2008). Here, we showed no changes in insulin during continuous 1 h of eVNS. Similarly, we have previously shown no significant changes in insulin during eVNS in normal rats during a glucose tolerance test (Payne et al., 2020). One explanation could be that the glucose lowering mechanisms of eVNS are independent of the insulin response. However, other studies have found insulin increases when VNS is applied to the distal (i.e., efferent) cut end of the cervical vagus nerve in normal rats (Meyers et al., 2016). Therefore, an alternative explanation is that the beta cell destruction from STZ injection compromises the stimulation‐induced release of insulin. We also report a decrease in glucagon levels that correlated with glycemic reduction, which suggests the mechanism of action could be through the suppression of glycogenolysis in the liver. It remains an open possibility that the rate of glycogenesis is directly mediated by the hepatic vagal efferent fibers (Niijima, 1985).

We observed no histopathological signs of Wallerian degeneration or changes in fascicle area of the implanted vagus nerve. Although there were no changes in fascicle area, we observed elongation of fascicles localized around electrodes with high‐frequency blocking stimuli (E3‐E4, 3 of 10). The elongation of tissue was often associated with a mature but benign, fibrotic response. However, evoked neural responses were still recorded in nerves that sustained morphological changes, indicating the health and functionality of the tissue. Furthermore, it was unlikely that the displacement of the nerve from the E3‐E4 electrodes impacted on the efficacy of therapy, as the mean daily glycemia of these affected animals (ID: 33, 34) decreased during acute eVNS (Figure 3a,b). This supports eVNS as safe for chronic use and could potentially be a clinically viable therapy of type 2 diabetes.

In conclusion, we have demonstrated for the first time in awake, metabolically compromised rats that eVNS applied to the abdominal vagus nerve is effective in reducing glycemia, an effect that is repeatable, chronic and persists despite the continued influence of disease promoting factors, such as a high‐fat diet. Future studies that have rigorous unstimulated controls should investigate the efficacy of eVNS in regulating hyperglycemia and reducing key blood glucose biomarkers (e.g., fasted blood glucose, hemoglobin A1c) over a long‐term 3‐month treatment period during which stimulation is applied during natural feeding times.

## CONFLICT OF INTEREST

All authors declare no conflict of interest, financial or otherwise.

## AUTHOR CONTRIBUTIONS

All listed authors made substantial, direct, and intellectual contributions to the study and manuscript.

## ETHICS STATEMENT

Procedures were approved by the St. Vincent's Hospital (Melbourne) Animal Ethics Committee (Project/approval number: 025–18) and complied with the Australian Code for the Care and Use of Animals for Scientific Purposes (National Health and Medical Research Council of Australia) and the Prevention of Cruelty to Animals (1986) Act.

## Data Availability

Our data are available in an external publicly shared folder in Dryad: https://doi.org/10.5061/dryad.cjsxksn72.
